# The influence of place on COVID-19 vaccine coverage in Alberta: A multilevel analysis

**DOI:** 10.1371/journal.pone.0276160

**Published:** 2022-10-14

**Authors:** Yuba Raj Paudel, Crystal Du, Shannon Elizabeth MacDonald

**Affiliations:** 1 School of Public Health, University of Alberta, Edmonton, Alberta, Canada; 2 Faculty of Nursing, University of Alberta, Edmonton, Alberta, Canada; Institute for Advanced Sustainability Studies, GERMANY

## Abstract

While there is evidence of urban/rural disparities in COVID-19 vaccination coverage, there is limited data on the influence of other place-based variables. In this cross-sectional study, we analyzed population-based linked administrative health data (publicly-funded health insurance database and province-wide immunization repository) to examine vaccination coverage for 3,945,103 residents aged 12 years and above in Alberta, Canada. We used multilevel logistic regression to examine the association of vaccination coverage with various place-based variables. Furthermore, we combined information on vaccine coverage and neighborhood level COVID-19 risk to categorize forward sortation areas (FSAs) into six categories. After 4 months of widely available COVID-19 vaccine, coverage varied widely between rural and urban areas (58% to 73%) and between geographic health authority zones (55.8% to 72.8%). Residents living in neighborhoods with lower COVID-19 disease incidence had the lowest vaccination coverage (63.2%), while coverage in higher incidence neighborhoods ranged from 68.3% to 71.9%. The multilevel logistic regression model indicated that residence in metro (adjusted odds ratio [aOR] 1.37; 95% CI: 1.31–1.42) and urban areas (aOR 1.11; 95% CI: 1.08–1.14) was associated with higher vaccine coverage than residence in rural areas. Similarly, residence in Edmonton, Calgary, and South health zones was associated with higher vaccine coverage compared to residence in Central zone. Higher income neighborhoods reported higher vaccine coverage than the lowest-income neighborhoods, and the highest COVID-19 risk neighborhoods reported higher vaccine coverage than the lowest risk neighborhoods (aOR 1.52; 95% CI: 1.12–2.05). In the first four months of wider vaccine availability in Alberta, COVID-19 vaccine coverage varied according to various place-based characteristics. Vaccine distribution strategies need to consider place-based variables for program prioritization and delivery.

## Introduction

Successful control of the COVID-19 pandemic depends on collective action, including rapid and widespread vaccination [1]. It is well known that vaccine coverage levels differ by individual-based variables (e.g., age, attitudes toward vaccines), but place-based variables also play a role [2].

Rural-urban residence is a variable commonly included in analyses of COVID-19 vaccination coverage. Studies from the USA have shown variability in vaccine coverage in rural versus urban regions. Data from April 2021 showed COVID-19 vaccine coverage among rural residents was 58.5% compared to 75.4% among urban residents [3], although individual-level characteristics were not considered. Another study found similar results, in addition to variation among rural counties [4].

The reasons for lower vaccine uptake in rural regions are multifaceted, as seen in a number of studies form the USA. One study suggested that the disparity was linked with lower access to health care in rural counties, as well as higher vaccine hesitancy and low-risk perception [3]. Another study showed that areas with slow vaccine rollout were more likely to be the regions with high barriers related to healthcare access and resources [1]. These areas were historically under-vaccinated, had irregular healthcare-seeking behaviour, and more socio-demographic barriers [1]. Their data also showed geographic clustering of barriers, with some counties exhibiting more barriers than others. Another study showed lower educational attainment was associated with low vaccination in rural areas [4]. Further, rural counties with farming and mining dependent economies had lower coverage than rural counties with recreation-dependent economies.

Understanding the association of place-based variables with vaccine coverage helps to identify community-level barriers for COVID-19 vaccine uptake that may inform decision makers to apply targeted measures. While rural-urban differences for COVID-19 vaccination are well described in the US literature, there are other less-studied factors related to place that may impact coverage. The role of neighborhood level COVID-19 risk, based on the proportion of COVID-19 positive population, on vaccine coverage has not been previously investigated. In this study, we examined the association between COVID-19 vaccination status and place-related variables, including rural-urban residence, geographic health zone, neighborhood income quintile, and neighborhood COVID-19 risk quintile, while also adjusting for individual-level demographic variables (age and sex).

## Materials and methods

### Setting

This study was conducted in Alberta, a western province of Canada with a population of 4.5 million. Alberta has universal health care insurance under which over 99% of Albertans are registered. Publicly funded COVID-19 vaccine was widely available throughout the province starting mid-May 2021 for all Albertans 12 years and older.

### Study design, and data sources

This was a population-based cross-sectional study using administrative data retrieved from the Alberta Ministry of Health data repositories. We used the Alberta Health Care Insurance Plan (AHCIP) quarterly population registry for the first quarter of 2021 to identify residents of the province. We used the Immunization and Adverse Reaction to Immunization (Imm/ARI) repository to determine COVID-19 vaccination status (as of August 31, 2021). Imm/ARI includes all records of COVID-19 vaccines administered in Alberta, regardless of provider. The total number of individuals who tested positive for COVID-19 was derived using laboratory data (as of August 31, 2021). We deterministically linked the databases using unique personal health numbers.

### Study cohort

Our cohort included all Alberta residents (as of first quarter of 2021) aged 12 years and above. Albertans <12 years were excluded because they were not eligible to receive the COVID-19 vaccine during the study period. We excluded First Nations residents of Alberta (since data for First Nations communities was not consistently submitted to Imm/ARI), Lloydminster residents (since vaccines are delivered by the neighbouring province), and those who left the province or died during the study period. S1 Fig presents sample selection flow chart.

### Outcome measure

We calculated ‘COVID-19 vaccination coverage’ as the proportion of eligible Alberta residents who received at least one dose of a COVID-19 vaccine as of August 31, 2021. We chose this time point to examine vaccination coverage during a time when vaccines were widely available.

### Exposure variables

#### Individual characteristics

We divided participant age into six categories, based on age categories used to prioritize COVID-19 vaccine eligibility in Alberta: 12–17 years, 18–29 years, 30–49 years, 50–64 years, 65–74 years, and 75 years and above. Biological sex at birth was categorized into male and female.

#### Place-based characteristics

Place-based variables, identified by complete 6-digit postal code, included: (1) rural-urban residence, categorized into metro and moderate metro, urban and moderate urban, and rural and remote rural, based on Alberta Health Services’ standard geographic areas classification system [5]. The classification is based on population size from 2016 Canadian census data and distance from the metro or urban centre (2) five geographic health zones of the province used for local health care program decision making (South, Calgary, Central, Edmonton, and North zones); (3) neighborhood income quintiles (Q1 indicating the lowest income neighborhood and Q5 indicating the highest income neighborhood), based on the 2016 Canadian census. We used neighborhood income quintile as a measure of socioeconomic status. Statistics Canada used the income per single-person Equivalent (IPPE) determined from the 2016 census to calculate neighborhood income quintile [6]. The IPPE was calculated as total income for an area divided by the Single Persons Equivalent (SPE). SPE reflects decreased costs per person when living two or more people in a family. Within each dissemination area, the average IPPE was calculated. Then, within each region (delineated by the census tract, the census agglomeration), dissemination areas were given a rank based on average IPPE and classified into quintiles. The household income quintiles and income cut-off values are community specific, reflecting the variations in housing and living cost across Canada. We linked the residential postal code of the person with the postal code-linking file available from Alberta Health to determine neighborhood income quintile for each participant. One place-based variable, (4) neighborhood COVID-19 risk quintile, was identified at the Forward Sortation Area (FSA) level, derived from the first 3 digits of the postal code. We collected data on COVID-19 positive population from provincial laboratory database. Only those with the confirmed case status were included in the analysis. Neighborhood COVID-19 risk quintile was defined based on the cumulative number of people that tested positive for COVID-19 as of August 31 2021 out of the total population of the FSA (expressed as proportion/10,000 population). Risk quintiles were defined as: >700 (risk quintile 1, includes 8 FSAs), 550–700 (risk quintile 2, includes 23 FSAs), 450–550 (risk quintile 3, includes 32 FSAs), 350–450 (risk quintile 3, includes 57 FSAs), <350 (risk quintile 5, includes 30 FSAs).

### Statistical analysis

We measured vaccination coverage by place-related variables: urban/rural place of residence, geographic health zone, neighbourhood income quintile, and neighborhood COVID-19 risk quintile. We used multilevel logistic regression to estimate effect sizes, adjusting for individual-level variables (sex and age), while accounting for clustering within each FSA. Before running multilevel logistic regression, we tested for multicollinearity among predictors, and no strong correlation was observed.

We performed analysis using GLIMMIX command in SAS according to the approach suggested by Ene et al with some adaptations [7]. First, we ran an empty unconditional model with no exposure variables included (Model 1). Next, we added variables defining individual-level characteristics (age categories and sex) into the model (Model 2). After this, place-based variables defined at the postal code level (urban/rural place of residence, geographic health zone, neighborhood income quintile) were included in the model (Model 3). Next, variables showing significant association in the fixed effects in model 3 were included in the random statements into the model (Model 4). Finally, fixed effects of the place-based variable defined at the FSA level was added to the model (Model 5).

The final model included variables at the individual level (variables defining characteristics of the individuals [age categories and sex]; place-based variables defined at the postal code [rural-urban place of residence, geographic health zone, neighbourhood income quintile], and a variable at the FSA level [neighborhood COVID-19 risk quintile]. To calculate intra-class correlation, we assumed that the dichotomous outcome variable (receipt of at least one dose of vaccine) came from a latent variable (continuous) with a level-1 residual which follows a logistic distribution having a mean equal to 0 and a variance equal to 3.29 [8]. Intra-class correlation (ICC) is a measure of correlation in an outcome variable among individuals living within a same unit (i.e., school, neighborhood). In other words, ICC indicates the amount of variation in vaccination coverage that is accounted for by variation between groups (between FSA variations) [9]. An ICC value of 0 indicates that all variability in vaccination lies within the FSA. Whereas an ICC value of 1 indicates all variability in vaccine coverage lies between FSAs (individuals within an FSA have the same vaccine coverage [10]. Literature suggests that chances of type I error (showing a significant effect, when in fact when no effect exists) increases with increasing value of ICC [10]. Therefore, hierarchically structured data need to be analysed using multilevel models.

We then combined information on vaccination coverage and neighborhood COVID-19 risk quintiles to define and map FSA categories on a map of the province, using QGIS software. We used the Alberta FSA boundary file (Forward Sortation Area Boundary File) published by Statistics Canada as a base map [11]. We performed statistical analysis using SAS 9.4 (SAS Institute Inc., Cary, NC) with a statistical significance set at p<0.05. The University of Alberta Health Research Ethics Board granted ethical approval for this study.

## Results

### Cohort characteristics

Of the total population included in this analysis (n = 3,945,103), nearly half (49.6%) were female. The majority (70%) lived in metropolitan areas (Table 1). By geographic health zones, Calgary (40%) and Edmonton Zone (32.7%) contained the majority of the population. Less than 10% of the total population lived in neighborhoods with the highest COVID-19 risk quintile; most of the population lived in neighborhoods with medium to low medium risk quintiles (60%).

**Table 1:** COVID-19 vaccination coverage by individual and place-based characteristics, as of August 31, 2021, in Alberta, Canada (N = 3,945,103).

Characteristics	Vaccinated	Unvaccinated	p value
	(≥ one dose)	(no dose)	
	% (n)	% (n)	
**Age category**			
12–17 years	62.9 (205,240)	37.1(120,827)	<0.0001
18–29 years	62.9 (424,326)	37.1 (249,843)	
30–49 years	64.4 (918,233)	35.6 (507,803)	
50–64 years	74.5 (642,084)	25.5 (219,683)	
65 to 74 years	81.1 (323,157)	18.9 (75,195)	
75 years and above	82.0 (212,087)	18.0 (46,625)	
**Sex**			
Females	71.3 (1,396,547)	28.7 (561,962)	<0.0001
Males	66.9 (1,328,580)	33.1 (658,014)	
**Place of residence**			
Metro	73.1 (2,010,401)	26.9 (738,732)	<0.0001
Urban	62.4 (299,103)	37.6 (179,973)	
Rural	58.0 (415,623)	42.0 (301,271)	
**Geographic health zone**			
South	63.4 (171,150)	36.6 (98,737)	<0.0001
Calgary	72.7 (1,148,512)	27.3 (432,228)	
Central	59.9 (241,388)	40.1 (161,526)	
Edmonton	72.8 (940,818)	27.2 (350,869)	
North	55.8 (223,259)	44.2 (176,616)	
**Neighborhood income quintile**			
Q5 (Highest income)	72.7 (595,519)	27.3 (223,305)	<0.0001
Q4	70.8 (586,582)	29.2 (242,565)	
Q3	69.6 (552,301)	30.4 (240,963)	
Q2	67.4 (530,707)	32.6 (257,121)	
Q1 (Lowest income)	64.2 (460,018)	35.8 (256,022)	
**Neighborhood COVID-19 risk quintile**			
R1 (Highest risk)	71.9 (237,315)	28.1 (92,763)	<0.0001
R2	68.3 (377,240)	31.7 (175,481)	
R3	71.5 (664,873)	28.5 (264,804)	
R4	70.0 (1,004,100)	30.0 (429,697)	
R5 (Lowest risk)	63.2 (441,599)	36.8 (257,231)	

### Vaccination coverage by place-related variables

The vaccine coverage in metro areas was 25 percentage point higher compared to rural areas and nearly 11 percentage point higher than in urban areas. Similarly, geographic health zones comprising of metro areas, i.e., Edmonton (72.8%) and Calgary (72.6%), had higher coverage compared to largely rural health zones, i.e., North (55.8%), Central (59.9%), and South (63.4%) zones. A clear gradient in vaccine coverage was observed by neighborhood income quintile. People living in neighborhoods with the lowest COVID-19 risk quintile had the lowest vaccination coverage (63.2%). Other neighborhood risk quintiles reported a similar vaccination coverage to each other ranging from 68.3% to 71.9%.

### Multilevel logistic regression

ICC from the unconditional model was 0.05 (ICC ranges from 0 to 1) indicating that there was a little variation in vaccination coverage between FSAs. The adjusted ORs from the final multilevel model (Table 2; S1 Table) showed that participants living in metro areas (aOR 1.37; 95% CI: 1.31–1.42) and urban areas (aOR 1.11; 95% CI: 1.08–1.14) had higher vaccination coverage compared to rural residents. People living in Calgary zone (aOR 1.26; 95% CI: 1.21–1.31), South zone (aOR 1.17; 95% CI: 1.12–1.22), and Edmonton zone (aOR 1.11; 95% CI: 1.05–1.18) had higher vaccination coverage compared to Central zone. People living in North zone (aOR-0.82; 95% CI: 0.77–0.86) had lower vaccine coverage than Central zone. People residing in the highest income neighborhoods were nearly twice as likely to get vaccinated than those living in the lowest income neighborhood (aOR 1.76; 95% CI: 1.60–1.93). Neighborhoods in middle-income quintiles (Q2, Q3, Q4) also had higher vaccination coverage than the lowest income quintile neighborhoods. People living in neighborhoods with the highest COVID-19 risk had higher vaccination coverage compared to those in the lowest risk neighborhoods (aOR 1.52; 95% CI: 1.12–2.05). Although other risk quintiles had a higher crude vaccination coverage than the lowest income quintile neighborhoods, the effect disappeared in the multivariable model (Table 2; S1 Table).

**Table 2:** Multilevel logistic regression analysis of factors associated with receipt of one dose of COVID-19 vaccine in Alberta, Canada^a^.

Characteristics	Model 2	Model 3	Model 4	Model 5
	AOR (95% CI)	AOR (95% CI)	AOR (95% CI)	AOR (95% CI)
**Age categories**				
12–17 years	Ref	Ref	Ref	Ref
18–29 years	1.00 (0.99–1.01)	1.01 (1.0–1.02)	1.00(0.94–1.06)	1.01 (0.89–1.14)
30–49 years	1.05 (1.04–1.05)	1.05 (1.04(1.06)	1.03 (0.97–1.09)	1.04 (0.92–1.18)
50–64 years	1.78 (1.77–1.79)	1.79 (1.78–1.81)	1.78 (1.67–1.88)	1.77 (1.56–2.01)
65 to 74 years	2.68 (2.65–2.71)	2.71 (2.68–2.74)	2.59 (2.44–2.75)	2.66 (2.35–3.02)
75 years and above	2.82 (2.79–2.86)	2.89 (2.86–2.93)	2.81(2.64–2.99)	2.88 (2.54–3.26)
**Sex **				
Male	Ref	Ref	Ref	Ref
Female	1.21 (1.21–1.22)	1.21 (1.21–1.22)	1.21 (1.20–1.23)	1.21 (1.20–1.23)
**Income Quintile**				
Q1 (Lowest income)	N/A	Ref	Ref	Ref
Q2	N/A	1.14 (1.13–1.15)	1.12 (1.07–1.17)	1.22 (1.11–1.33)
Q3	N/A	1.28 (1.27–1.29)	1.26 (1.21–1.32)	1.37 (1.25–1.49)
Q4	N/A	1.42 (1.41–1.44)	1.40 (1.34–1.46)	1.50 (1.37–1.64)
Q5 (Highest income)	N/A	1.67 (1.65–1.68)	1.62 (1.55–1.70)	1.76 (1.60–1.93)
**Geographic health zone**				
Central zone	N/A	Ref	Ref	Ref
South zone	N/A	0.93 (0.90–0.96)	0.90 (0.86–0.94)	1.17 (1.12–1.22)
Calgary zone	N/A	1.19 (1.16–1.23)	1.23 (1.19–1.27)	1.26 (1.21–1.31)
Edmonton zone	N/A	1.03 (0.98–1.08)	1.04 (0.98–1.10)	1.11 (1.05–1.18)
North zone	N/A	1.17 (1.12–1.23)	1.02 (0.96–1.07)	0.82 (0.77–0.86)
**Place of residence**				
Rural	N/A	Ref	Ref	Ref
Metro	N/A	1.28 (1.24–1.33)	1.25 (1.20–1.30)	1.37 (1.31–1.42)
Urban	N/A	1.01 (0.98–1.03)	1.00 (0.98–1.03)	1.11 (1.08–1.14)
**Neighborhood COVID-19 risk level**				
R5 (Lowest risk)	N/A	N/A	N/A	Ref
R1 (Highest risk)	N/A	N/A	N/A	1.52 (1.12–2.05)
R2 (Higher risk)	N/A	N/A	N/A	1.14 (0.93–1.39)
R3 (Medium risk)	N/A	N/A	N/A	1.15 (0.96–1.38)
R4 (Lower risk)	N/A	N/A	N/A	1.12 (0.96–1.32)

^a^ Notes

Model 1-Empty unconditional model with no exposure variables, not shown in the table

Model 2. Variables defining individual-level characteristics of the individuals (age categories and sex) added into the Model 1.

Model 3. Place-based variables defined at the postal code level (urban/rural place of residence, geographic health zone, neighborhood income quintile) were added into the Model 2.

Model 4. Variables showing significant association in the fixed effects in model 3 were included in the random statements into the Model 3.

Model 5 (final model): Place-based variable defined at the FSA level (Neighborhood COVID-19 risk level) was added to the Model 4.

**Fig 1:**
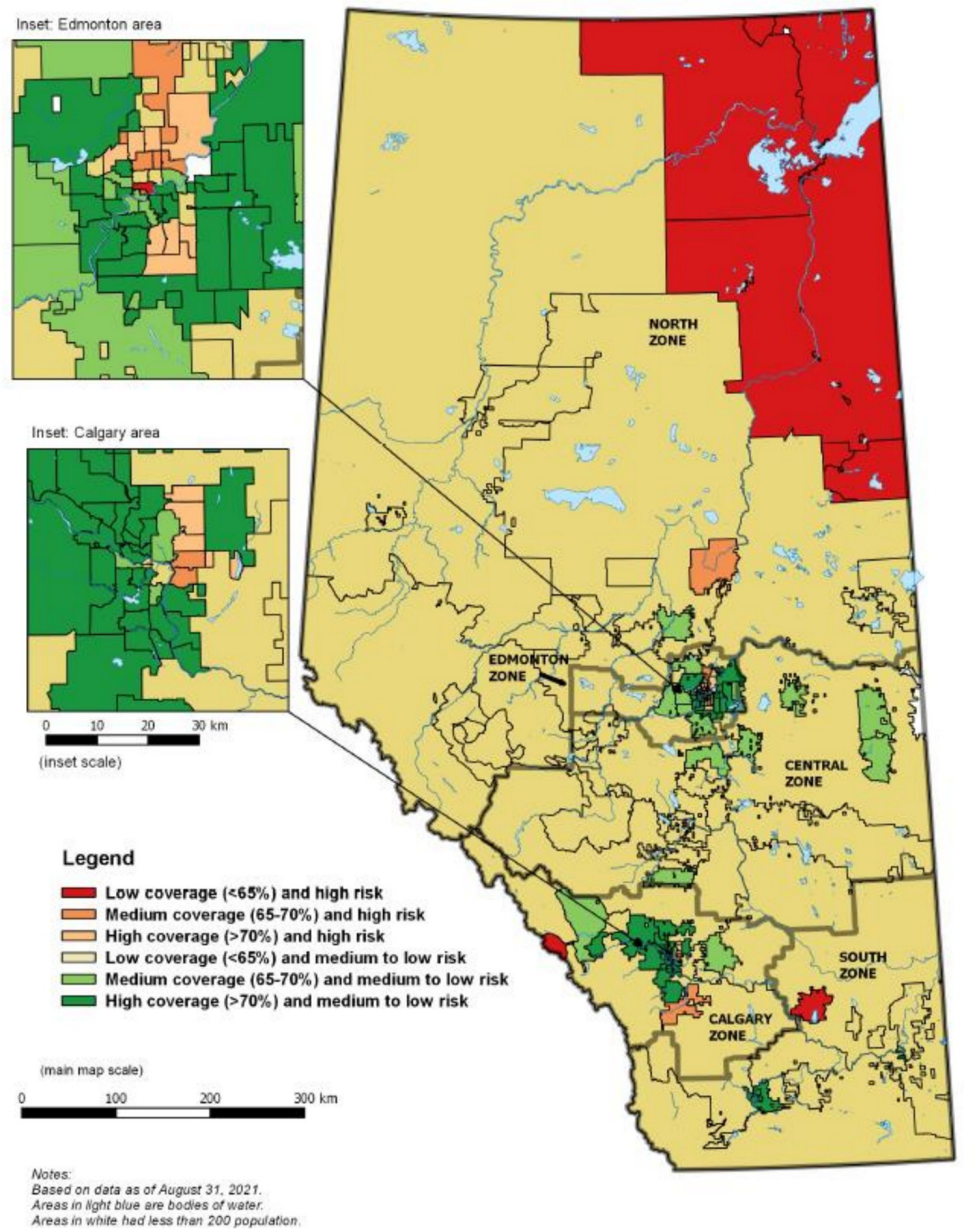
Map of Alberta forward sortation areas (FSAs) showing COVID-19 vaccination coverage and neighborhood risk quintile. *Note*: This map was created using Alberta FSA boundary file as a base map by linking with vaccine coverage and neighborhood level COVID-19 risk quintile using QGIS software. Use of this product is governed by the Statistics Canada Open License Agreement which provides permission for a “worldwide, royalty-free, non-exclusive license to use, reproduce, publish, freely distribute, or sell the information” [12].

Six categories of FSAs were mapped (Fig 1), representing the following combination of vaccination coverage and neighborhood COVID-19 risk quintiles:

1. Low coverage (<65%) and high risk (risk quintile 1 or 2)- includes 10 FSAs
2. Medium coverage (65–70% coverage) and high risk (risk quintile 1 or 2)—includes 8 FSAs
3. High coverage (>70% coverage) and high risk (risk quintile 1 or 2)—includes 13 FSAs
4. Low coverage (<65%) and medium to low risk (risk quintile 3 to 5)—includes 38 FSAs
5. Medium coverage (65–70% coverage) and medium to low risk (risk quintile 3 to 5)- includes 27 FSAs
6. High coverage (>70% coverage) and medium to low risk (risk quintile 3 to 5)- includes 54 FSAs

Mapping vaccination coverage by neighborhood risk quintile indicated that the majority of category 6 FSAs (high coverage and medium to low risk) were located in three cities (Calgary, Edmonton and Lethbridge). The Northeast part of Calgary and Edmonton included category 2 and 3 FSAs, defined as high risk and high to medium coverage. Category 5 FSAs, defined as medium coverage and medium to low risk, were in the outskirts of these three cities. Most category 4 FSAs, defined as low coverage and medium to low risk, were in the rural areas of each zone. Category 1 FSAs, defined as low coverage and high risk, were in industrial zones of North zone (Fort McMurray, Fort McKay), South zone (Brooks), and Calgary zone (Banff).

## Discussion

### Summary

We found that place-based variables had a significant association with COVID-19 vaccination coverage after adjusting for age categories and sex. Multilevel logistic regression analysis revealed that there was higher vaccine coverage among people living in metro and urban areas compared to those in rural areas; those in higher income neighborhoods compared to poorest lowest income neighborhoods; and those in South zone, Calgary, and Edmonton compared to those living in Central zone, while those in North zone had lower coverage. People living in the highest COVID-19 risk neighborhoods had higher vaccination coverage than those living in the lowest risk neighborhoods.

### Interpretation

Our analysis showed that vaccination coverage in Alberta, as of August 2021, ranged from 58% in rural areas to 73% in large metropolitan regions, including Calgary and Edmonton. This is consistent with US findings, where vaccination coverage was consistently higher in urban centres than in neighbouring rural regions [3]. Decreased access to vaccine appointments, and challenges with vaccine supply in rural Alberta during initial stages of vaccine roll-out might have contributed to low vaccine uptake. Reportedly, vaccine supply in rural Alberta was not consistent, making management of appointments challenging, often resulting in cancelled appointments [13]. Correspondingly, the North, South, and Central health zones, which consist mainly of rural regions, reported lower vaccine uptake than Edmonton and Calgary Health Zones. Some of the regions with the lowest coverage had few pharmacies offering the vaccine with minimal access to government-run clinics [14]; rural residents often had to travel to nonadjacent regions to access vaccination sites [9]. In addition, rural residents have been shown to have lower levels of educations and may be more vaccine hesitant, which may contribute to lower coverage [15–17]. Consistent with a US-based study [4], we found that coverage also varied between rural areas. The North zone had the lowest vaccination coverage, with only 55.8% having at least one dose. A US-based study found that disparities between rural counties was attributed to type of industry, with mining-dependent economies having lower vaccination coverage in comparison to recreational ones [4].

The lower income neighborhoods reported lower vaccination coverage compared to the highest income neighborhoods. There was an apparent stepwise pattern in the crude vaccination coverage (Table 1), as well as in the multivariable model (Table 2). An Ontario-based study also showed that residents living in neighborhoods with high material deprivation had a low one-dose, two-dose, and booster dose coverage [18]. News reports have suggested that transportation, internet literacy, and language barriers are significant for low-income Albertans to receive vaccination [15].

We hypothesized that areas with the highest COVID-19 risk quintile would have lower vaccination coverage. Similar work from Ontario showed that vaccination coverage was lower in areas defined as highest risk for COVID-19 infection [16]. Interestingly, in our study, we found that vaccination coverage was higher in areas defined as the highest COVID-19 risk quintile in comparison to the coverage in lowest risk quintile neighborhoods, with some regional variation. We found that most of the category 2 and 3 FSAs (highest COVID-19 risk neighborhood with high to medium coverage) lay in Northeast Calgary, and Northeast Edmonton. Although low vaccine turnouts occurred at the beginning of the pandemic in these areas and surrounding communities, there were focused efforts to improve vaccination by launching mobile clinics, targeted messaging, and engaging with community leaders, which might have improved vaccine uptake [17]. Additionally, Northeast Calgary (40%) and Northeast Edmonton has a high proportion of immigrant population; it is known that a significant proportion of immigrants are working as front-line workers, who may find social distancing difficult [19, 20]. We have previously shown that immigrants in Alberta have higher vaccination coverage than the non-immigrant population, which may also reflect this high-risk employment [21].

Furthermore, areas defined as high COVID-19 risk quintile and low coverage were in the Northwest of Calgary zone (Banff), Northwest of South zone (Brooks), and Northern Alberta (Fort McMurray, Fort McKay) (Fig 1). These areas have meat processing plants (Brooks), oil sands industry (Fort McMurray, Fort McKay), and recreational industry (Banff), which reported outbreaks at the initial stage of the pandemic [22–24]. Following the outbreaks, the infection might also have spread to nearby communities through workers and their families, which led to a high number of cases [25]. It was reported that these areas faced a vaccine supply issue at the initial stage of the pandemic but were later targeted by improved vaccine delivery strategies. Notably, some of these areas (e.g. Banff) currently have a high vaccination coverage (>80%) [26].

### Implications

These findings suggest that vaccine supply in rural areas needs to be timely and consistent. Focusing on largely rural health zones (North zone, South zone, and Central zone) and rural areas within other health zones may be an optimal strategy for vaccine promotion and delivery. Similarly, residents living in low-income neighborhoods also require extra support. Furthermore, industrial workers who work in closed spaces with less chances of physical distancing need to be prioritized for vaccination [27]. Therefore, vaccine distribution and promotion strategies should not only look at individual risk factors but also to where people work and live [28].

### Strengths and limitations

This is one of the few studies conducted assessing the relationship of place-related variables with COVID-19 vaccine uptake. We used a population-based registry and immunization repository to assess vaccination status, making our findings representative of the entire province. We used multilevel analysis to account for the hierarchical nature of our data. We used measures that utilized community specific data to categorize neighborhood level income quintile (median income of the community considering household size) and COVID-19 risk quintile (proportion of people who tested positive in a community), ensuring the reliability of the measures. However, our study has some limitations. We defined neighborhood risk quintiles based on the cumulative number of positive cases in each FSA. The high incidence may be the result of a big outbreak at the beginning of the pandemic and may not reflect the high-risk status in the later period. We could not include additional individual-level (e.g. educational status, political ideology, vaccine attitudes), household level variables (e.g. household size/income), and other place-based variables (e.g. distance to nearest clinic/pharmacy), due to lack of such data. Our estimated coverage is lower than coverage posted on the Alberta Health public website due to potential inclusion of people who departed the province without reporting to the health insurance program in the denominator [29]. We assumed that there is no systematic difference in departure pattern by place-based variables we have included in this analysis. If an individual received a vaccine after departing the province in other jurisdictions, they are misclassified as unvaccinated in the current analysis. Our analysis was aimed at assessing place-based distribution of vaccination and does not imply causality. Furthermore, the findings may not be generalizable to other jurisdictions.

## Conclusion

People living in rural areas, those in Central, South and North zones, those in the lowest income neighborhoods, and those in the lowest COVID–19 risk quintile neighborhoods had lower vaccine coverage at time of this study. Vaccine delivery policies and implementation strategies needs to focus on these areas to improve timely vaccination.

## Supporting information

S1 Fig Sample selection flow chart. (TIFF)

S2 Fig Flow diagram showing database linkage. (TIFF)

S1 Table Factors associated with receipt of one dose of COVID-19 vaccine in Alberta, Canada. (TIFF)
